# Interleukin gene expression in broiler chickens infected by different *Escherichia coli* serotypes

**DOI:** 10.14202/vetworld.2021.2727-2734

**Published:** 2021-10-24

**Authors:** Reham Elnagar, Rasha Elkenany, Gamal Younis

**Affiliations:** Department of Bacteriology, Mycology, and Immunology, Faculty of Veterinary Medicine, Mansoura University, Mansoura 35516, Egypt.

**Keywords:** broiler chicken, *Escherichia coli*, experimental infection, histopathology, interleukins

## Abstract

**Background and Aim:**

*Escherichia coli* is the cause of avian colibacillosis, a significant threat to the poultry industry and public health. Thus, this study investigated the prevalence of *E. coli* in diseased chicken broilers, pathological effects of these bacteria, and interleukin (IL) gene expression of different serotypes of *E. coli* (O78, O26, O44, and O55) on experimentally infected chickens.

**Materials and Methods:**

A total of 295 organ samples (liver, lungs, heart, and spleen) from 59 diseased broiler chickens were used for conventional identification of *E. coli*. Chickens were orally infected with one of the following *E. coli* serotypes (O78, O26, O44, or O55) and examined for clinical signs, mortality, macroscopic and microscopic lesions, and IL gene expression using real-time quantitative polymerase chain reaction.

**Results:**

*E. coli* was isolated from 53.2% of broiler chicken organs with a high prevalence in lungs (26.1%). The most prevalent serotypes were O78, O26, O44, O55, O157, and O127 prevalence of 27.8, 22.2, 16.7, 16.7, 5.6, and 5.6%, respectively. In the experimental design, five groups (G1-G5) of birds were established. G1 served as the negative control group, while G2-G5 were challenged orally with *E. coli* O78, O26, O55, or O44, respectively. Chickens infected with *E. coli* O78 or O26 showed significant clinical signs in comparison to the other infected birds. Mortality (13.3%) was only observed in birds infected with *E. coli* O78. Necropsy of dead birds after *E. coli* O78 infection showed pericarditis, enteritis, airsacculitis, and liver and lung congestion. More severe histopathological changes were observed in intestines, spleen, liver, and lung from chickens infected with either *E. coli* O78 or O26 than for birds infected with other serotypes. On the 2^nd^ day post-infection, *E. coli* challenge, particularly with *E. coli* O78, displayed significantly upregulated levels of ileal IL-6 and IL-8, but ileal IL-10 level tended to be downregulated in comparison to the control group.

**Conclusion:**

This study assessed the application of cytokines as therapeutic agents against infectious diseases, particularly colibacillosis.

## Introduction

*Escherichia coli* (*E. coli*) is a typical microorganism in chicken intestinal tracts and trachea to a lesser degree [1]. Even common “non-pathogenic” strains of *E. coli* cause infection in animals, humans, birds, and weakened or immunocompromised hosts or when gastrointestinal barriers are breached. Further, some strains of *E. coli* invade the body and cause avian colibacillosis, a fatal systemic disease [2]. Signs of colibacillosis in poultry include infection of yolk sacs, omphalitis, hepatitis, pericarditis, septicemia, polyserositis, enteritis, cellulitis, respiratory tract infection, and salpingitis [3]. Poultry produces its immune cells and antibodies to provide protection during disease recovery [4].

*E. coli* displays molecular patterns, such as lipopolysaccharides (LPS) and flagellin, on its surface that interacts with toll-like receptors on the surface of macrophage, resulting in the secretion of interleukins (IL-1, IL-6, and IL-8) [5-7]. ILs are polypeptides produced by cells involved in immune and inflammatory responses. ILs activate and modulate other cells and tissues [8]. ILs produced by birds are poorly understood in terms of structure and function. However, recent progress in the field of avian immunology and genetics now recognizes a variety of ILs, mostly in chickens. Relatively few recombinant cytokines or monoclonal antibodies against avian cytokines have been produced, but available technologies, such as real-time quantitative polymerase chain reaction (PCR), allow quantification of messenger RNA expression from cytokine genes without the use of proteins or antibodies [9]. These tools open a whole new world of possibilities for determining the levels of ILs in illnesses, leading to a better understanding of pathogenesis and immune mechanisms. ILs also show a potential for controlling avian infectious diseases and are being examined as new curative agents. ILs may also serve as vaccine adjuvants, activating the immune system to encourage an effective defense response [9].

ILs, for example, IL-6, are secreted proteins involved in recruiting and controlling cells in both natural and acquired immunities. Such cytokines are needed for successful host immune responses to pathogens. Chicken IL-6 and its role in pro-inflammatory response are confirmed [10]. IL-6 aids short-term protection against infection or damage by alerting the immune system to the source of inflammation. However, illness arises from improper control of this molecule. Pro-inflammatory cytokines, such as IL-6, control the immune response by stimulating the proliferation and differentiation of leukocytes that kill pathogens [11]. The pro-inflammatory chemokine, IL-8, is a chemoattractant, drawing heterophils to the site of infection and triggering a fast local inflammatory reaction [12]. IL-8 is known for chemotactic activity toward heterophils and macrophages in chickens [13]. Further, macrophage activation stimulates the secretion of the crucial anti-inflammatory cytokine IL-10 that keeps the immune system balanced by suppressing excessive pro-inflammatory cytokine production [14]. IL-10 acts as an inflammation feedback factor, allowing modulation of the immune response [15]. IL-10 promotes Th2-like immune responses, primarily through suppressing Th1 and pro-inflammatory cytokines [16]. These anti-inflammatory properties, including inhibition of macrophage function, can make the host more susceptible to bacterial infections.

A study by Li *et al*. [17] reported identification and virulence of *E. coli*; however, limited research concentrates on the interaction of *E. coli*, particularly *E. coli* serotypes O26 and O44, with host innate immune response. Consequently, the primary goal of this research was the assessment of pathological and immunological influences of different *E. coli* serotypes in broiler chickens. The current study was designed to (1) determine *E. coli* prevalence in diseased broiler chickens; (2) describe impacts of infection by different serotypes of *E. coli* clinical signs, mortalities, macroscopic lesions, and histopathology; (3) and identify the effects of infection by different serotypes on IL gene expression (IL-6, IL-8, and IL-10) in broiler chickens using reverse transcription-quantitative PCR (RT-qPCR).

## Materials and Methods

### Ethical approval

Animal Experiments and Ethics Committee, Faculty of Veterinary Medicine, Mansoura University approved all protocols and humane euthanasia (physical method).

### Sample collection, study period, and location

A total of 295 organ samples (liver, lungs, heart, and spleen) from fifty-nine 21-50-day-old broiler chickens were separately collected in the period from April to October 2019. All chickens displayed signs of disease and were obtained from 11 poultry farms situated in various geographic areas in Dakahlia and Sharqia Governorates in Egypt. The chickens exhibited diarrhea, loss of appetite, coughing, and sneezing. Ascites, pericarditis, perihepatitis, airsacculitis, and peritonitis were the most common lesions observed during necropsy. Samples were separately collected in sterile polyethylene bags, placed immediately on ice, and transferred to the laboratory for bacteriological examination.

### Isolation and identification of *E. coli*

A loopful from each organ was cultured on MacConkey’s agar (Oxoid, UK) plates incubated aerobically at 37°C for 24 h. Suspected pink colonies were picked up, streaked, and incubated overnight at 37°C on eosin methylene blue (EMB) agar (Oxoid). Biochemical tests, such as catalase, oxidase, urease, methyl red, Voges–Proskauer, citrate utilization, indole, and triple sugar iron agar, were used to identify suspected *E. coli* colonies [18]. Slide agglutination with polyvalent and monovalent fast diagnostic sets of *E. coli* antisera (Denka Seiken Co., Ltd., Japan) was used to identify serotypes of *E. coli* isolates [19].

### Experimental design

#### Preparation of E. coli inocula

*E. coli* serotypes O78, O26, O55, and O44 obtained from local cases of avian colibacillosis as described above were enriched in trypticase soy broth (Oxoid) and incubated at 37°C for 24 h. The surface spread method was used to determine viable cells per mL of trypticase soy broth [20]. *E. coli* suspensions containing 10^9^ CFU/mL were prepared as previously described by Ateya *et al*. [21]. Infection with *E. coli* serotypes was achieved by oral administration of 0.5 mL of bacterial suspension to 7-day-old chicks using a syringe attached to a polyethylene tube. Birds were given a similar amount of sterile phosphate buffer saline as a negative control.

#### Experimental chicks

The experiment used 75 healthy Hubbard broiler chicks obtained from a commercial hatchery when 1 day old. Broiler chicks were divided into five groups of 15 birds at random. All birds were supplied with a balanced commercial starter till day 14 throughout the experiment. Fresh and clean drinking water was supplied *ad libitum*. Chicks were housed in a clean well-ventilated, previously fumigated room. The first group (G1) served as a standard negative control group, the second group (G2) was infected by *E. coli* O78, the third group (G3) by *E. coli* O26, the fourth group (G4) by *E. coli* O55, and the fifth group (G5) by *E. coli* O44.

#### Clinical symptoms, mortality, and macroscopic lesions

Birds in each group were monitored twice a day for clinical symptoms and mortality. *E. coli* colonization in visceral organs and gross lesions was by gathering, necropsying, and taking specimens of dead and sacrificed birds.

#### E. coli reisolating from internal organs

Two chickens per group were randomly selected and sacrificed on days 7 and 14 post-inoculation for reisolating *E. coli* from visceral organs. Liver, spleen, lungs, and cecum samples from these birds were streaked on EMB agar and incubated at 37°C for 18-24 h. Suspected *E. coli* colonies were identified using morphological, biochemical, and serological methods, as mentioned before.

#### Histopathological examination

On day 14, samples from liver, spleen, lung, intestine, and heart were fixed in 10% neutral buffered formalin. Hematoxylin and eosin stained 5 μm thick paraffin sections were examined microscopically [22].

### Collection of ileum sample

On day 2, five birds from each group were sacrificed at random for collecting ileal samples. Ileal specimens were aseptically washed in phosphate-buffered saline, snap frozen in RNA later (Qiagen, Germany) solution, and stored at −80°C for later quantification of gene expression.

### RT-qPCR

IL gene expression (IL-6, IL-8, and IL-10) in ileal tissue was quantified using RNA extraction used an RNeasy Mini Kit (Catalogue no.74104, Qiagen). A 30 mg of tissue sample was homogenized and processed as described by the manufacturer. IL-6, IL-8, and IL-10 mRNA were amplified and quantified using a real-time PCR machine (Stratagene MX3005P, Agilent Technologies Company, USA). Primer sequences are listed in Table-1 [23,24]. The housekeeping gene 28S rRNA was used as a constitutive control for normalization. The reaction mixture volume was 25 μL made up with 12.5 μL 2x QuantiTect Probe RT-PCR Master Mix (catalog No.204443, Qiagen), 0.5 μL of 20 pmol solution of each primer, 0.125 μL probe (30 pmol), 0.25 μL QuantiTect RT Mix (RevertAid Reverse Transcriptase) (Qiagen), 8.125 μL RNase-free water (Sedico, Egypt), and 3 μL template RNA. The real-time PCR cycling conditions were reverse transcription (50°C, 30 min), primary denaturation (94°C, 10 min), and then amplification (40 cycles) with secondary denaturation (94°C, 15 s), annealing, and extension (60°C, 1 min). Amplification curves and Ct values were determined using Stratagene MX3005P software, Agilent Technologies Company). The Ct value of each sample was compared with the positive control as described for the “DDCT” method [25].

**Table-1:** Oligonucleotide primers and probes used in real-time PCR.

Gene	Primer sequence	Reference

	(5’- 3’)	
*IL-6*	GCTCGCCGGCTTCGA	
	GGTAGGTCTGAAAGGCGAACAG	[23]
	(FAM) AGGAGAAATGCCTGACGAAGCTCTCCA (TAMRA)	
*28S rRNA*	GGCGAAGCCAGAGGAAACT	
	GACGACCGATTTGCACGTC	
	(FAM) AGGACCGCTACGGACCTCCACCA (TAMRA)	
*IL-8*	GCCCTCCTCCTGGTTTCAG	
	TGGCACCGCAGCTCATT	
	(FAM) TCTTTACCAGCGTCCTACCTTGCGACA (TAMRA)	
*IL-10*	CATGCTGCTGGGCCTGAA	[24]
	CGTCTCCTTGATCTGCTTGATG	
	(FAM) CGACGATGCGGCGCTGTCA (TAMRA)	

PCR=Polymerase chain reaction

### Statistical analysis

Data were analyzed using one-way analysis of variance with the statistical program SPSS, version 17.0 (SPSS Inc., Chicago, IL, USA). Means of ileal gene expression levels were compared at p<0.05 using Tukey’s test for statistical significance.

## Results

### *E. coli* prevalence in sick broiler chicken

*E. coli* was isolated from 32 of 59 broiler chickens (54.2%). A total of 157 (53.2%) isolates of pathogenic *E. coli* were obtained from 295 samples. These isolates were identified morphologically and biochemically from chicken organs. The highest incidence of *E. coli* was detected in lungs 41/157 (26.1%), followed by liver 40/157 (25.5%), heart 33/157 (21.1%), and spleen 24/157 (15.3%). Serological identification of 18 biochemically identified *E. coli* strains showed six serotypes (Table-2). *E. coli* serotype O78 was predominant (27.8%), followed by O26 (22.2%), O44 and O55 (16.7% each), and O157 and O127 (5.6% each).

**Table-2:** Serotyping of *E. coli* isolates from diseased chicken (n=18).

Serotype	Types of samples	No. of isolates	Percentage
O78	Lungs (n=2), liver, heart, spleen	5	27.8
O44:K74	Spleen, heart, liver	3	16.7
O55:K59	Heart, lung (n=2)	3	16.7
O26:K60	Lung, heart (n=2), spleen	4	22.2
O157:K-	Lung	1	5.6
O127	Liver	1	5.6

*E. coli=Escherichia coli*

### Experimental infection results

#### Clinical signs, mortality, and macroscopic lesions

All broiler chickens inoculated with *E. coli* serotypes O78, O26, O55, or O44 were inspected. Ruffled feathers, inappetence, respiratory manifestations, sitting on hocks, and diarrhea (yellow to whitish) were the most common signs observed in experimentally infected birds. Clinical signs were more severe in birds in G2 (O78 infected) and G3 (O26 infected) than in birds in G4 (O55 infected) and G5 (O44 infected). Signs observed in birds in G2 were more severe than signs recorded from other birds. Mortality was only observed in G2 birds (13.3%; 2/15); no mortality was recorded in other chickens throughout the experimental period. Pericarditis, enteritis, airsacculitis (cloudy to fibrinous exudate in air sacs), and liver and lung congestion were seen during necropsy of dead birds in G2. No clinical symptoms or deaths were observed in the control birds (G1).

#### E. coli reisolation from internal organs

Recovery of *E. coli* from internal organs of different groups was performed. *E. coli* strains were reisolated from visceral organs (liver, spleen, lung, and cecum) of chickens in all experimentally infected birds (G2-G5) on days 7 and 14. No *E. coli* was recovered from any control birds.

#### Histopathological findings of internal organs

**Figure-1:**
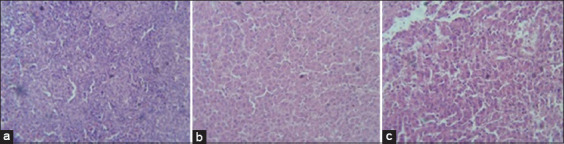
Photomicrograph of sections from liver of broiler chicks on 7 days post-infection with *Escherichia coli* stained with H&E. (a) Histological picture of liver shows normal picture in G1 (control group); (b) interstitial, hemorrhages, portal tract mononuclear cell infiltration, congestion, and focal feathery degeneration in G2; (c) severe hemorrhages, less inflammation, interstitial hemorrhages, portal tract mononuclear cell infiltration, congestion, and focal feathery degeneration in G3.

**Figure-2:**
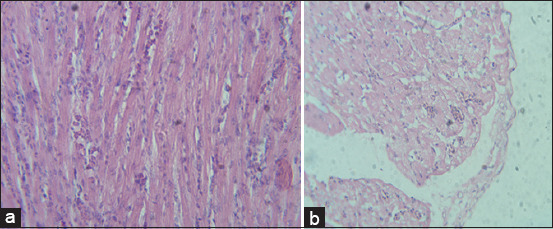
Photomicrograph of sections from heart of broiler chicks on 7 days post-infection with *Escherichia coli* stained with H & E. (a) Histological picture of heart shows normal picture in G1 (control group); (b) mild thickening of pericardium, degeneration of myocardium, and no significant inflammatory cell infiltrate in G2 and G3.

**Figure-3:**
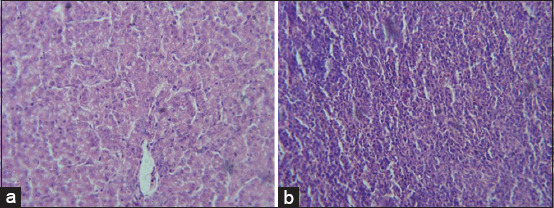
Photomicrograph of section from spleen of broiler chicks on 7 days post-infection with *Escherichia coli* stained with H & E. (a) Histological picture of spleen shows normal picture in G1 (control group); (b) congestion, focal necrosis, and dilated sinusoids by inflammatory cells infiltrate in G2 and G3.

**Figure-4:**
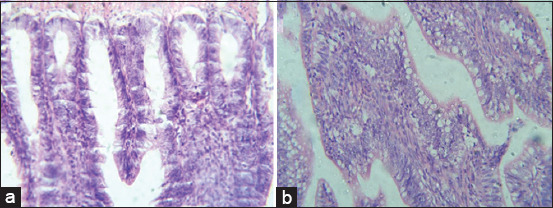
Photomicrograph of sections from intestine of broiler chicks on 7 days post-infection with *Escherichia coli* stained with H & E. (a) Histological picture of heart shows normal picture in G1 (control group); (b) sloughing of epithelium and inflammatory cell infiltration in G2 and G3.

**Figure-5:**
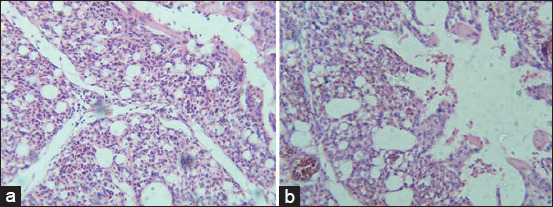
Photomicrograph of sections from lung of broiler chicks on 7 days post-infection with *Escherichia coli* stained with H & E. (a) Histological picture of lung shows normal picture in G1 (control group); (b) severe congestion, hemorrhages, and inflammatory cellular infiltrate in G2, G3, and G5.

Histopathological lesions observed in livers of G2 chickens were inflammation, interstitial hemorrhages, portal tract mononuclear cell infiltration, congestion, and focal feathery degeneration (Figure-1b). Livers from G3 birds showed similar lesions but with more hemorrhages and less inflammation (Figure-1c) in comparison to control (Figure-1a). Lesions in hearts from G2 and G3 were mild thickening of pericardium, degeneration of myocardium, but no significant inflammatory cell infiltration (Figure-2b) in comparison to control (Figure-2a). Congestion, focal necrosis, and dilated sinusoids with inflammatory cells were observed in the spleen in birds in G2 and G3 (Figure-3b) in comparison to control (Figure-3a). In the intestine, sloughing of intestinal epithelium and inflammatory cell infiltration was severe in chickens in G2 and G3 (Figure-4b) but less serious in birds in G4 in comparison to control (Figure-4a). In the lungs, severe congestion, hemorrhage, and moderate inflammatory cellular infiltration were observed in birds in G2, G3, and G5 (Figure-5b) G4 in comparison to control (Figure-5a).

#### E. coli infection upregulates expression of IL-6, IL-8, and IL-10

**Figure-6:**
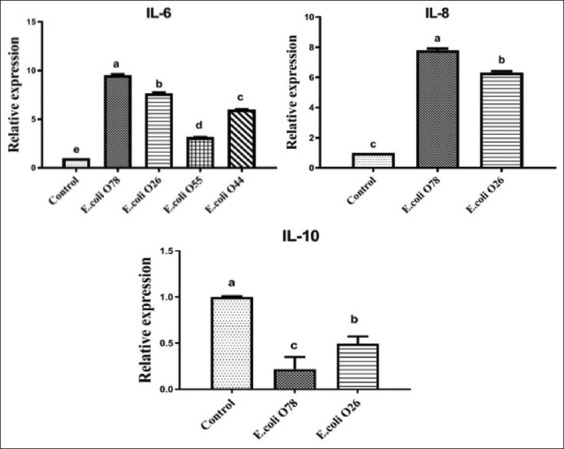
Reverse transcription-polymerase chain reaction analyses for the expression of pro-inflammatory cytokine (IL-6, IL-8, and IL-10) in the ileum of birds challenged with different serotypes of *Escherichia coli* (O78, O26, O55, and O44). Data were expressed as mean±standard error. The different letters indicate a significant difference (p<0.05) between experimental groups.

Relative quantitative RT-qPCR was used to assess relative gene expression of pro-inflammatory cytokines (IL-6, IL-8, and IL-10) in the ileum of birds following *E. coli* challenge on 2 days after infection. In contrast to the control group, *E. coli* challenge elevated mRNA expression of ileal IL-6 and IL-8 but appeared to lower ileal IL-10 expression (Figure-6). The highest significant elevation in mRNA expression of IL-6 was recorded in birds from G2. Other chickens (G1, G3, G4, and G5) showed significant downregulation in ileal IL-6 (p<0.05). IL-6 gene expression in G2 showed the greatest effect, a 9.5-fold increase over controls. The smallest effect was observed in birds from G4, a 3-fold change (Figure-6). Chickens from G2 also showed higher ileal IL-8 levels than birds from G3 and G1. However, G2 ileal IL-10 level was significantly lower.

## Discussion

Primary infection in the respiratory tract can be caused by pathogenic *E. coli*. Such infection may result in systemic infection and appearance of a disease known as colibacillosis in chickens [26]. Lower hatching rates, egg production, and inhibition of growth along with increased mortality occur due to colibacillosis, which is currently the main cause of economic losses in the poultry industry worldwide [27]. The incidence of *E. coli* isolated from samples of Egyptian broiler chickens that showed clinical signs of colibacillosis was 53.2%. Similarly, the previous studies showed a high incidence of *E. coli* (52.3%, 53.8%, and 57.3%) at different localities in Egypt [28-30]. Furthermore, *E. coli* was isolated from 53.4% of the internal organs of ill broiler chickens in Jordan [31]. Conversely, a lower incidence of *E. coli* in chickens was previously reported in the range of 26.9-36.5% [32-34]. Pilling of *E. coli* in aerosols in chicken barns allows birds to inhale *E. coli* and supports a high disease incidence in chicken broilers.

*E. coli* was found in a variety of internal organs, with high rates of isolation from lungs (26.1%) and liver (25.5%) [33,35]. The isolation of *E. coli* from several organs is a sign of intestinal *E. coli* invasion. High isolation rates from lungs and liver may be attributed to an initial respiratory tract infection that spreads to other internal organs [36].

The serotyping analysis that distinguishes pathogenic strains based on surface antigens is a more accurate identification method. Serogroup O is one basic diagnostic for classifying pathogenic *E. coli* strains. Serological identification showed that serotype O78 (27.8%) was predominant followed by O26 (22.2%) than other serotypes. O78 was the dominant serotype in Egypt among other serogroups (O1, O26, O2, O127, O91, and O153) isolated from broiler chicken viscera [28]. Broiler chickens infected with *E. coli* in China and Jordan showed that O78 was the most prevalent serotype [31,37]. O26 was identified in the present investigation from chickens suffering from septicemia. This serogroup is not common in chickens but may be transmitted from other animals to chickens raised nearby. Thus, raising chickens at a distance from other animals may be important for preventing the transmission of *E. coli* isolates among livestock.

Ruffled feathers, decreased appetite, respiratory symptoms, sitting on hocks, and yellow to whitish diarrhea were the most commonly noted signs in experimentally infected birds. In birds infected with *E. coli* O78 and O26 (G2 and G3), clinical manifestations were more severe, with 13.3% mortality. This result is consistent with a previous study [38] that reported mouth breathing, sneezing, ruffled feathers, weights loss, diarrhea, loss of appetite, diarrhea, and mortality (30%) in chickens infected with *E. coli* O78. Another study indicated depression with whitish diarrhea in broiler chickens infected experimentally with *E. coli* O78 [21,39]. Birds exhibited a 12.82% mortality after 48 h among birds challenged with *E. coli*. Further, postmortem examination of the dead birds in chickens infected with *E. coli* O78 (G2) in the present study showed pericarditis, enteritis, airsacculitis, and liver and lung congestion. A similar investigation also reported congestion in different organs, as well as fibrin accumulation in the liver (bread and butter appearance) and heart [40].

Histopathological lesions varied from mild to severe, reflecting virulence and pathogenicity. Chickens infected with *E. coli* O78 or O26 displayed more severe histopathological changes in the liver, spleen, intestine, and lung samples than birds infected with other serotypes. In contrast, only mild lesions were exhibited in heart tissues. Some findings may be due to infection-induced immunosuppression and stress [41]. A previous study by Shah *et al*. [40] also reported fibrinous pericarditis, myocarditis, fibrinous perihepatitis, hepatitis, fatty changes in hepatocytes, interstitial pneumonia, necrosis, and depletion of lymphocytes in spleen, consistent with these findings. Finally, inflammation, congestion, and degenerative changes in the liver, heart, splenic cells, and intestine with sloughing of epithelium were reported [42].

ILs are extracellular signaling molecules that transmit information between cells for modulating immune responses as part of both natural and adaptive immunities [8]. Relative RT-qPCR is the most accurate method for the detection and quantification of gene expression in cells and tissues for low abundance mRNA due to its high sensitivity, good reproducibility, and wide quantification range [43]. Gene expression in this study showed a relationship between pro-inflammatory cytokine levels (IL-6, IL-8, and IL10) and the challenge induced by various *E. coli* serotypes in broiler chickens. Upregulation of mRNA expression of ileal IL-6 and IL-8 genes, but downregulation of ileal IL-10 expression followed *E. coli* challenge. These changes occurred early after infection (day 2), and IL-6 and IL-8 likely participate in host immune response to pathogens [41,44]. Both cytokines are critical for initiating an acute-phase immune response against invading pathogens and triggering a variety of immune cells, such as T cells and macrophages [9]. A significant increase in *IL-6* and *IL-8* gene expression in the ileum of birds infected with *E. coli* O78 (G2) was seen compared to other chickens. Upregulation may be a result of greater pathogenicity of *E. coli* serotype O78 compared to other examined serotypes. Thus, *E. coli* O78 might overstimulate the immune system to a greater extent than other tested serotypes. Inoculation with *E. coli* induced secretion of pro-inflammatory cytokines IL-1b, IL-6, and IL-8, especially in splenic cells and pulmonary tissue [21,45,46]. Overall, small increases in pro-inflammatory cytokines may reflect the age of chicks. Natural resistance to infection with *E. coli* had developed in chicks by the age of 3 weeks [47].

Further, phagocytic cells of the natural immune system secrete IL-10 due to *E. coli* infection. In this study, anti-inflammatory cytokine (*IL-10*) gene expression was reduced after exposure to *E. coli* O78 or O26 that was compatible to previous studies[21,48]. Expression of IL-10 was suppressed after exposure to LPS [49,50]. When broilers were nourished with mannanoligosaccharide and then challenged with *E. coli*, ileal IL-10 expression was higher than when they were challenged with *E. coli* only [46]. Furthermore, in chickens infected with *E. coli*, a large elevation in liver IL-10 occurred [51]. Differences in gene expression among studies could be due to different challenge approaches and measuring methods.

## Conclusion

This is the first study to demonstrate the impact of avian *E. coli* serotypes O44 and O26 on the pathological status and IL gene expression in broiler chickens. A high prevalence of *E. coli* in broiler chicken that might be associated with major economic losses was found. Experimental infection with different serotypes of *E. coli* connected adverse effects with a shift in the equilibrium of cytokines. IL-6 and IL-8 are upregulated and IL-10 downregulated. This imbalance causes inflammation-induced damage in several internal organs. Further studies might clarify the utility of cytokines as therapeutic agents against infectious diseases, particularly colibacillosis. In addition, the application of control and preventive measures is highly recommended for poultry farms to limit bacterial diseases, such as colibacillosis, that are associated with major economic losses and public health hazards.

## Authors’ Contributions

GY and RaE: Designed the experiment, supervised the study, and drafted the manuscript. RE: Collected the samples and carried out the practical part. All authors read and approved the final manuscript.
